# The Size of the Foveal Avascular Zone Is Associated with Foveal Thickness and Structure in Premature Children

**DOI:** 10.1155/2019/8340729

**Published:** 2019-07-02

**Authors:** Akiko Miki, Yuko Yamada, Makoto Nakamura

**Affiliations:** Department of Surgery, Division of Ophthalmology, Kobe University Graduate School of Medicine, Kobe 6500017, Japan

## Abstract

**Purpose:**

To investigate the foveal avascular zone (FAZ) in the eyes of patients with a history of retinopathy of prematurity (ROP) using optical coherence tomography angiography (OCTA) and to identify associated clinical factors.

**Patients and methods:**

Overall, 14 children with a history of laser treatment for ROP, 17 children born prematurely without a history of ROP, and 41 age-matched children born at full-term (age range 7–14 years) were included. OCTA was conducted on an area measuring 3 × 3 mm in the central macula. The area of FAZ in the superficial layer was measured. Foveal thickness (FT), ganglion cell complex thickness, and the presence of inner retinal layer (IRL) at the fovea were evaluated.

**Results:**

There were significant differences in FT and FAZ size among patients (*P* < 0.001). The eyes of patients that had been treated for ROP showed the smallest FAZ and greatest FT. Univariate analyses demonstrated that the area of FAZ was not correlated with visual acuity (*P*=0.078) but with gestational age (GA) (*P*=0.001), birth weight (*P*=0.013), the presence of IRL (*P* < 0.001), and FT (*P* < 0.001). Multivariate regression analyses showed that the area of FAZ was significantly associated with GA, the presence of IRL, and FT (*P*=0.03, *P*=0.01, *P* < 0.001, respectively).

**Conclusion:**

The eyes of preterm children had small FAZ, and this reduction in area was associated with greater FT, the presence of IRL, and lower GA.

## 1. Introduction

The fovea has no retinal vasculature and contains a depression with no inner retinal layers. Recent studies [1–3] using optical coherence tomography (OCT) have demonstrated that the eyes of patients with a history of retinopathy of prematurity (ROP) have foveal dysplasia. Lack of centrifugal movement of the inner retina contributes to abnormal foveal morphology, which may affect the foveal avascular zone (FAZ).

OCT angiography (OCTA) is a useful tool for noninvasive visualization of blood vessels. Recently, the resolution of OCTA images has been reported to be higher than that of conventional fluorescein angiography (FA) images [4]. Although a previous study evaluated the area of FAZ in cases with a history of ROP using FA, OCTA can provide more precise measurements of FAZ.

Previous reports have demonstrated that premature children who underwent ROP treatment have smaller FAZ and thicker foveal thickness [5, 6]. However, the conclusion differed in these papers regarding the relationship between visual acuity and FAZ. It has recently been reported that the area of FAZ was associated with foveal retinal structure. Thus far, there were no reports describing the relationship between inner retinal architecture at the fovea and FAZ in premature children. Moreover, both eyes from one patient were assessed in these papers, though ROP was affected by various systemic conditions. In this study, we assessed one eye from one patient. We aimed to quantify the area of FAZ using OCTA in the eyes of patients with a history of laser treatment for ROP and compare these measurements to those taken from the eyes of preterm children without a history of ROP and control subjects and to identify the clinical factors associated with these findings, including premature status, retinal structure, and visual acuity.

## 2. Materials and Methods

This study was approved by the institutional review board at Kobe University Graduate School of Medicine and was conducted in accordance with the Declaration of Helsinki. Written informed consent was obtained from the caregivers of all subjects. Patients were recruited from the Department of Ophthalmology at Kobe University Hospital in Japan. Children born at full-term (gestational age (GA) > 37 weeks), who were age-matched to children born prematurely, were recruited as control subjects.

The criteria for screening were as follows: infants whose GAs were less than 34 weeks, infants whose birth weights were less than 1800 g, infants who suffered from severe birth asphyxia, and infants who required the supplementation of high-density oxygen for long term were screened for ROP. Treatment for ROP was performed according to the recommendations of the Early Treatment for ROP Study [7]. Patients who received surgical treatment or were administered intravitreal antivascular endothelial growth factor were excluded from this study.

All patients with a history of laser treatment for ROP (treated-ROP group, *n*=14; age range 8–12 years), children born prematurely but without a history of ROP (preterm without ROP group, *n*=17; age range 7–15 years), and age-matched controls (control group, *n*=41; age range 7–14 years) underwent ophthalmic examinations, including best-corrected visual acuity measurements, subjective refraction measurements, and slit-lamp biomicroscopy of the fundi.

An IOLMaster biometer (Carl Zeiss, Dublin, CA) was used to measure the axial length of the eye. The corneal curvature was measured in the horizontal and vertical medians using an auto keratometer RC5000 (Tomey, Phoenix, AZ). A Cirrus HD-OCT (Carl Zeiss) was used for identifying the presence of IRL using 5-line scan protocol and for measuring the foveal thickness (FT) using the macular cube protocol (200 × 200) and the ganglion cell-inner plexiform layer thickness with ganglion cell analysis. OCTA was conducted using a Cirrus Angioplex (Carl Zeiss). A centralized scan area measuring 3 × 3 mm was selected in the superficial layer, and OCTA images of this area were generated automatically. FAZ in the superficial layer in masked OCTA images was measured by graders using ImageJ software (ImageJ 1.48v; National Institutes of Health, Bethesda, MA, USA). The set-scale parameter of the software was used to define a 1024-pixel width as 3 mm. Binary images were created through thresholding, and edge points along the centerline of the vessels were manually selected.

All assessments were performed on both eyes. However, in most patients, the results of the right eye were used for analyses. Left-eye measurements were conducted for some patients owing to the poor-quality OCTA images of the right eye. For statistical analyses, the decimal visual acuity was converted to logarithm of the minimum angle of resolution (logMAR) units.

Differences in variables among the three groups (treated-ROP, preterm without ROP, and control groups) were analysed using Kruskal–Wallis test. Differences in variables between the two groups (treated-ROP group and preterm without ROP group) were analysed using Mann–Whitney *U* test. The correlations between FAZ and clinical parameters were analysed using Spearman correlation.

Statistical analyses were performed using SPSS (Version 25.0, IBM Corp., Armonk, NY). A *P* value of <0.05 was considered to be statistically significant.

## 3. Results

The baseline characteristics of the treated-ROP, preterm without ROP, and control groups are presented in Table 1.

Measurements from one eye each of 72 subjects (treated-ROP group, *n*=14; preterm without ROP group, *n*=17; and control group, *n*=41) were included in this study. All patients in the treated-ROP group had received laser photocoagulation treatment in the eye that was measured. No significant differences were present in age, gender, or axial length. However, there were significant differences with respect to visual acuity, refractive error, and mean corneal curvature among groups (*P*=0.001, *P*=0.02, and *P*=0.02, respectively). There were also significant differences in GA and birth weight (BW) between the treated-ROP and preterm without ROP groups (*P* < 0.001). OCTA findings are presented in Table 2.

**Figure 1:**
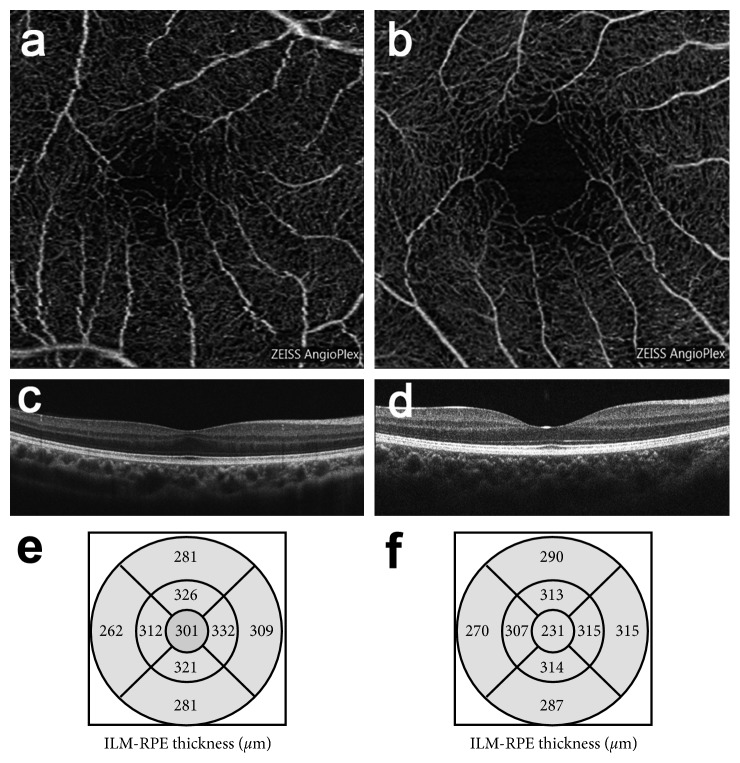
Representative images of optical coherence tomography angiography (OCTA) and OCT in patients with a history of laser treatment for retinopathy of prematurity (treated-ROP) and age-matched controls. (a, b). Comparison of the foveal avascular zone (FAZ) in the superficial vascular plexus assessed using OCTA ((a) treated-ROP group; (b) control group). (c, d). Comparison of the foveal structure ((c) IRL (+), treated-ROP group; (d) IRL (−), control group). (e, f). Retinal thickness of the Early Treatment Diabetic Retinopathy Study areas ((e) treated-ROP group; (f) control group).

The smallest FAZ area was measured in the treated-ROP group (0.14 ± 0.11 mm^2^) compared with the preterm without ROP (0.32 ± 0.23 mm^2^) and control groups (0.36 ± 0.10 mm^2^; *P* < 0.001; Table 2, Figure 1). Additionally, the FT was found to be the greatest in the treated-ROP group (279.00 ± 14.69 *μ*m) compared with the preterm without ROP group (252.94 ± 29.97 *μ*m) and control group (241.49 ± 15.19 *μ*m; *P* < 0.001; Table 2, Figure 1).

Next, the factors associated with the FAZ area were investigated, and only preterm children (treated-ROP and preterm without ROP groups) were included in these analyses. Univariate analysis showed that the area of FAZ was significantly correlated with GA (*P*=0.001), BW (*P*=0.013), the presence of IRL at the fovea (*P* < 0.001), and FT (*P* < 0.001). The visual acuity and refractive error tended to be associated with the area of FAZ; however, no significant differences were noted (*P* = 0.078 and 0.074, respectively; Table 3).

**Figure 2:**
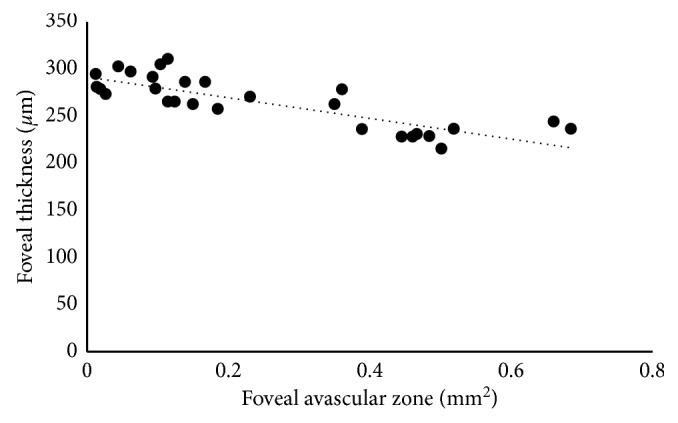
Correlation between the area of the foveal avascular zone (FAZ) and foveal thickness. The area of the FAZ is inversely correlated with foveal thickness (*r* = 0.80, *P* < 0.001).

In addition, following multiple regression analyses, the FAZ area was found to be significantly associated with GA (*P*=0.03), the presence of IRL at the fovea (*P*=0.01), and FT (*P* < 0.001). The FAZ area was negatively correlated with FT (*P* < 0.001, *r* = 0.80; Figure 2) and was positively correlated with GA and the presence of IRL at the fovea (*P*=0.03, *r* = 0.65 and *P*=0.01, *r* = 0.66, respectively).

## 4. Discussion

Using conventional fluorescein angiography, Mintz-Hittner et al. [8] were the first to describe a small or nonexistent FAZ in the eyes of patients with a history of ROP and document that the area of FAZ was correlated with GA and BW. Consistent with the results of this previous study, our study employed the use of OCTA to determine that the area of FAZ was smallest in patients with ROP. However, no absence of the FAZ was found in this group. This result may be associated with the relatively small number of participants assessed in this study. However, it is possible that the difference may be owing to the variation in the resolution of OCTA and FA images.

OCTA is a commercially available tool for noninvasive visualization of blood vessels. A better visualization of the vascular architecture of the eye is obtained using this technique than that obtained using conventional FA. Additionally, the method offers the benefit of clear assessment of FAZ without the potential for dye leakage. Assessment of *en face* images created using OCT is another new approach that can be used to noninvasively visualize FAZ. Using *en face* images obtained via spectral domain OCT (SD-OCT), Yanni et al. [9] reported that a smaller FAZ was present in preterm children. However, for assessment of the FAZ area, images obtained by OCTA are of higher quality than those obtained by SD-OCT.

FT was found to be the greatest in the eyes of patients with ROP treatment, and this finding is consistent with that of previous reports [1, 3]. During fovea development, ganglion cells and inner nuclear layers migrate peripherally, and outer segments of cones migrate toward the center [10, 11]. The development of the macula begins at approximately 25 weeks of gestation and continues until 3–4 years of age. The adverse effects of premature birth may disrupt this development process, resulting in a thickened fovea.

Multiple regression analysis revealed that GA is associated with the size of FAZ. Previous studies have suggested that the mechanism behind a small or absent FAZ in preterm children is related to the process of vascular remodeling of the fovea [7, 12]. In the human retina, blood vessels begin to form at the disc at approximately 14 weeks of gestation and reach the nasal margin of the incipient foveal pit by 25–26 weeks of gestation [13]. The fovea remains avascular throughout development [14, 15]. The presence of a factor that negatively affects angiogenesis is suggested to determine the discontinuation of vascular development in the fovea. FAZ forms at 37 weeks of gestation and continues to undergo remodeling up to approximately 15 months after birth [13, 14]. A previous study demonstrated that a small or absent FAZ was found in all preterm infants who had a GA of ≤30 weeks, and a normal FAZ was found in all infants who had a GA of ≥36 weeks [8]. On the basis of these results, it was suggested that changes in retinal arterial oxygen (Pa0_2_) levels after premature birth were associated with the factor that negatively affects angiogenesis, resulting in a small or absent FAZ. Premature babies born before 28 weeks of gestation require mechanical ventilation. The time between foveal vascular development and the environmental change experienced after birth may affect the development of FAZ. In the current study, the average GA of the treated-ROP group was approximately 26 weeks, whereas that of the preterm group without previous ROP was around 33 weeks.

Via multiple regression analysis, FT has been determined to be significantly correlated with FAZ. In previous study using retinal sections obtained from a monkey, researchers demonstrated that the avascular region was defined before the start of the foveal depression formation [16]. There are likely to be developmental interactions between the processes of FAZ formation and foveal depression with regard to timing. Hence, the abnormal formation of FAZ may directly or indirectly influence the development of the foveal depression.

Recently, Kulikov et al. [17] showed that FAZ area correlates with the area of absence of the inner nuclear layer. In ROP eyes, the inner nuclear area often remains at the fovea. Therefore, we investigated the association between FAZ and the presence of IRL. We found a significant correlation between FAZ and the status of IRL. This means that not only foveal thickness but foveal structure at the fovea also defines the FAZ. Since the retina requires the blood supply, small FAZ may contribute to the lack of inner retinal development.

In this study, FAZ was found not to be associated with visual acuity. Dubis et al. [18] demonstrated the relationship between the area of FAZ and degree of foveal pit excavation using normal subjects aged between 18 and 67 years. Marmor et al. [19] investigated the retinal function using adaptive optics and obtaining central multifocal electroretinograms in subjects without a foveal pit and suggested that the foveal pit is of no functional importance. In premature infants, the development of the inner and outer segments of photoreceptors was more delayed compared with that in full-term infants, but migration toward the center continued after birth [20]. The outer retina may be less affected by changes in retinal PaO_2_ compared with the inner retina with respect to development. Of note, the finding that FAZ is not associated with visual acuity might be associated with the exclusion criteria of this study. Subjects with low visual acuity were excluded because high-resolution imaging was difficult owing to poor fixation and nystagmus.

The current study has some limitations. The study included a limited number of patients, and no premature children with a history of regressed ROP were assessed. Future studies with a larger sample size will be needed to understand the clinical significance of a restricted FAZ in premature children.

## 5. Conclusions

In conclusion, this study of patients with ROP determined that the eyes of preterm children have a small FAZ, and the area of FAZ is associated with GA, FT, and the presence of IRL.

## Figures and Tables

**Table 1:** Baseline characteristics of patients with retinopathy of prematurity, preterm children without retinopathy of prematurity, and control subjects.

	Treated-ROP (*n*=14)	Preterm without ROP (*n*=17)	Control (*n*=41)	*P* value
Age (years)	10.0 ± 1.57	10.0 ± 2.87	9.41 ± 2.40	0.26^a^
Sex (male/female)	8/6	6/11	12/29	0.19^a^
Visual acuity (logMAR)	−0.038 ± 0.093	−0.14 ± 0.064	−0.15 ± 0.085	0.001^a^
Refractive error (D)	−3.79 ± 4.11	−0.31 ± 5.15	−0.17 ± 1.82	0.02^a^
Keratometry	7.52 ± 0.18	7.69 ± 0.22	7.69 ± 0.20	0.02^a^
Axial length (mm)	23.03 ± 2.01	23.38 ± 2.63	23.04 ± 1.11	0.94^a^
Gestational age (weeks)	26.86 ± 2.31	33.41 ± 4.20	—	<0.001^b^
Birth weight (g)	870.29 ± 213.61	1925.76 ± 688.40	—	<0.001^b^

Abbreviations: ROP, retinopathy of prematurity. ^a^Kruskal–Wallis test; ^b^Mann–Whitney *U* test.

**Table 2:** OCTA and OCT measurements in patients with retinopathy of prematurity and control subjects.

	Treated-ROP (*n*=14)	Preterm without ROP (*n*=17)	Control (*n*=41)	*P* value
FAZ superficial layer (mm^2^)	0.14 ± 0.11	0.32 ± 0.23	0.36 ± 0.10	<0.001
GCL thickness (*μ*m)	72.43 ± 19.6	77.00 ± 14.85	83.56 ± 4.23	0.13
Foveal thickness (*μ*m)	279.00 ± 14.69	252.94 ± 29.97	241.49 ± 15.19	<0.001
IRL at the fovea (+/−)	11/3	3/14	0/41	<0.001

Abbreviations: FAZ, foveal avascular zone; GCL, ganglion cell-inner plexiform layer; ROP, retinopathy of prematurity.

**Table 3:** Univariate and multivariate regression analysis of factors correlated with FAZ.

	Univariate coefficient	*P* value	Multivariate coefficient	*P* value
Age (years)	−0.21	0.256		
Sex (male = 1, female = 2)	0.196	0.291		
Visual acuity (logMAR)	−0.326	0.078		
Refractive error (D)	0.325	0.074		
Axial length (mm)	−0.272	0.139		
Keratometry	−0.068	0.718		
Gestational age (weeks)	0.546	0.001	0.69	0.03
Birth weight (g)	0.44	0.013	−1.43	0.17
GCL thickness (*μ*m)	0.263	0.153		
Foveal thickness (*μ*m)	−0.797	<0.001	−4.24	<0.001
IRL at the fovea (yes = 1, no = 2)	0.598	<0.001	0.43	0.01

Abbreviations**:** GCL, ganglion cell-inner plexiform layer; INL, inner nuclear layer.

## Data Availability

The data used to support the findings of this study are available from the corresponding author upon request.
